# 901. The Impact of Cost-Free On-Site Influenza Point of Care Antigen Testing on Influenza Detection in Nursing Homes

**DOI:** 10.1093/ofid/ofad500.946

**Published:** 2023-11-27

**Authors:** Yasin Abdul, Ed Davidson, Kevin McConeghy, Kaley Hayes, Lisa Han, Melissa LaMantia, Elie Saade, David Canaday, Stefan Gravenstein

**Affiliations:** Brown University, Providence, Rhode Island; Insight Therapeutics, LLC, Norfolk, Virginia; Providence VAMC, Providence, RI; Brown University, Providence, Rhode Island; Insight Therapeutics, LLC, Norfolk, Virginia; Insight Therapeutics, LLC, Norfolk, Virginia; University Hospitals, Cleveland, Ohio; VA Northeast Ohio Healthcare System, Cleveland, Ohio; Brown University, Providence, Rhode Island

## Abstract

**Background:**

Influenza point of care (POC) testing can support rapid influenza detection and outbreak management in nursing homes (NHs). We hypothesize cost-free on-site access to influenza POC tests increases POC test use and influenza detection. A current study positions us to evaluate this hypothesis.

**Methods:**

A prospective cohort of U.S. NHs enrolled in a comparative effectiveness trial of baloxavir versus oseltamivir for outbreak management through 2 influenza seasons, 2020-2022 (NCT05012189). We compared study NHs provided with influenza POC tests for use when clinically indicated to non-study facilities participating in weekly National Healthcare Safety Network (NHSN) reporting for occurrence of influenza outbreaks. For study facilities, we received reports upon influenza detection and directly surveyed them for POC use and influenza incidence. We compared study NHs and other NHSN NHs for reported influenza case rates.

**Results:**

We recruited 586 facilities with an average of 89 long-stay residents and 120 Medicare certified beds which reported 185 new influenza activity in 159 facilities. Study test use frequency was reported (N=250), mean use was 28 tests per facility whether or not influenza was detected. Upon influenza detection, 52% of NHs initiated antiviral treatment or chemoprophylaxis on 1 or more residents. Study NHs reported 201 cases with a cumulative incidence of 3.7 cases per 1000 resident days versus 2,124 cases (1.7 per 1000) in non-participating NHs in the same period. Non-participating U.S. NHs had fewer beds (76 long-stay residents), 106 Medicare certified beds and less than half the rate of outbreak detection from study facilities.

**Conclusion:**

NHs that participated in our prospective study reported influenza disproportionately more than other NHs reporting to NHSN. We interpret that the availability of easy-to-use and freely accessible rapid POC influenza tests positions NHs to detect better and manage influenza outbreaks. The ease of access to POC on-site tests may play a significant role and become a best practice approach in this positive outcome to improve influenza detection and support early intervention.

**Disclosures:**

**Yasin Abdul, MD**, Genentech: Grant/Research Support **Ed Davidson, PharmD, MPH**, Genentech: Grant/Research Support **Kevin McConeghy, PharmD**, Genentech: Grant/Research Support|Pfizer: Grant/Research Support|Sanofi-Pasteur: Grant/Research Support|Seqirus: Grant/Research Support **Kaley Hayes, PharmD, PhD**, Sanofi Aventis: Grant/Research Support **Lisa Han, MPH**, Genentech: Grant/Research Support **Melissa LaMantia, MA**, Genentech: Grant/Research Support **Elie Saade, MD**, Envision Pharma: Speaker, Presenter|Johnson and Johnson: Speaker, Travel, Lodging|Protein Sciences Corp: Grant/Research Support|Sanofi Pasteur: Speaker, Travel, Lodging **David Canaday, MD**, Pfizer: Grant/Research Support **Stefan Gravenstein, MD, MPH**, CDC: Grant/Research Support|Genentech: Advisor/Consultant|Genentech: Grant/Research Support|GSK: Advisor/Consultant|GSK: Honoraria|Janssen: Advisor/Consultant|Janssen: Honoraria|NIH: Grant/Research Support|Pfizer: Grant/Research Support|Pfizer: Honoraria|Sanofi: Advisor/Consultant|Sanofi: Grant/Research Support|Sanofi: Honoraria|Seqirus: Grant/Research Support|Seqirus: Honoraria

